# Effects of gut microbiota on the susceptibility of ischemic stroke in mice

**DOI:** 10.1128/msystems.00149-26

**Published:** 2026-07-10

**Authors:** Guangyan Wu, Huidi Wang, Qianyi Zhou, Jingxiang Fu, Feng Zhang, Zhuo Duan, Shuo Wang, Jielin Huang, Hongwei Zhou, Zhenchao Ma, Yan He, Jia Yin, Kaiyu Xu

**Affiliations:** 1 Microbiome Medicine Center, Department of Laboratory Medicine, Zhujiang Hospital, Southern Medical University 70570, Guangzhou, China; 2 Huzhou Central Hospital, Fifth School of Clinical Medicine of Zhejiang Chinese Medical University 70571, Huzhou, China; 3 Guangdong Provincial Clinical Research Center for Laboratory Medicine, Guangzhou, China; 4 State Key Laboratory of Muti-organ Injury Prevention and Treatment, Southern Medical University 70570, Guangzhou, China; 5 Key Laboratory of Mental Health of the Ministry of Education, Guangzhou, China; 6 Department of Neurology, Nanfang Hospital, Southern Medical University 70570, Guangzhou, China; APC Microbiome Ireland, Cork, Ireland

**Keywords:** gut microbiota, ischemic stroke, susceptibility, fecal microbiota transplantation

## Abstract

Ischemic stroke is a highly prevalent disease with limited therapeutic options, and emerging evidence suggests that the gut microbiota influences stroke pathophysiology. However, whether the gut microbiota affects individual susceptibility to ischemic stroke remains unclear. Here, middle cerebral artery occlusion was performed to stratify mice into ischemic stroke-sensitive (SEN: reaching humane endpoints within day 1) and ischemic stroke-resistant (RES: surviving to day 7) groups based on post-stroke survival. SEN mice exhibited more severe brain injury than RES mice, accompanied by increased systemic inflammation and elevated intestinal permeability. Fecal microbiota from SEN or RES donors was transplanted into antibiotic-treated recipients. Mice receiving SEN microbiota developed significantly worse outcomes compared with those receiving RES microbiota. Multi-omic analyses of cecal contents, including 16S rRNA gene sequencing and liquid chromatography-mass spectrometry/mass spectrometry-based untargeted metabolomics, identified reduced butyrate-producing bacteria and altered glutathione metabolism as potential contributors to stroke susceptibility. Collectively, these findings demonstrate that gut microbiota and microbial metabolites modulate susceptibility to ischemic stroke.

**IMPORTANCE:**

The role of the gut microbiota in determining susceptibility to ischemic stroke has remained poorly defined. This study demonstrates that microbiota dysbiosis and metabolite alterations functionally increase vulnerability to stroke injury, highlighting the gut microbiome as a potential target for risk stratification and preventive interventions. Modulating the gut microbiota may therefore represent a novel strategy for reducing stroke susceptibility.

## INTRODUCTION

In the past 30 years, the annual incidence cases of stroke and deaths due to stroke have increased dramatically, representing the third leading cause of death and the fourth leading cause of disability-adjusted life years globally (1, 2). Stroke mortality is projected to increase by 50% in the next three decades, because of the aging population and rising prevalence of metabolic diseases (2). Among stroke types, that is, ischemic stroke and hemorrhagic stroke, ischemic stroke accounts for approximately 75%–85% of all cases of stroke worldwide (3, 4). Metabolic disorders such as hypertension, type 2 diabetes, hyperlipidemia, and so on, predispose humans to ischemic stroke (5). More importantly, stroke outcome and clinical manifestations are highly heterogeneous across individuals. The severity of acute ischemic stroke is assessed by the National Institutes of Health Stroke Scale score, ranging from 0 to 42 points, with higher values indicating greater severity (6). Previous studies suggest that various factors, including genetics, sex, race (7–9), and more direct factors such as admission hyperglycemia, region of brain infarction (10), even seasonal variation, and socioeconomic status are associated with the severity of stroke (11, 12).

In recent years, increasing evidence has proven an intricate role of the gut microbiota in stroke, implicating a potential therapeutic target for stroke (13–15). Indeed, gut microbiota dysbiosis and altered bacteria-derived metabolites, such as short-chain fatty acids, bile acids, and trimethylamine N-oxide, have been observed in ischemic stroke patients (16–19). Furthermore, the causal relationship between gut microbiota and stroke injury has been verified by fecal microbiota transplantation (FMT) in animal studies (20–22). Our previous study found that the gut microbiota *per se* contributes to stroke injury and post-stroke cognitive function in stroke mice (23, 24). However, there are discrepancies and contradictory results between different studies regarding the key gut microbiota that contribute to stroke injury.

In the current study, using a middle cerebral artery occlusion (MCAO) model of ischemic stroke, we aimed to investigate the intestinal microbiome and metabolome that contribute to susceptibility to stroke injury and identify key microbiota and metabolites that may serve as biomarkers for stroke severity and potential therapeutic targets for intervention.

## MATERIALS AND METHODS

### Mice

Eight-week-old specific pathogen-free (SPF) male C57BL/6J mice (GemPharmatech Co., Ltd., China) were used as experimental animals and acclimated to the animal facility for 1 week. Mice were accommodated in an SPF facility with a 12 h/12 h light-dark cycle, an ambient temperature of 20°C–25°C, and humidity levels ranging from 30% to 70%. All mice had free access to food and water.

### Cerebral ischemia and reperfusion model

The model of focal cerebral ischemia was induced by transient MCAO using an intraluminal filament as previously described (24). Briefly, mice were anesthetized by intraperitoneal injection of 1.2% tribromoethanol (0.02 mL/g of body weight). During the study period, the body temperature of mice was meticulously regulated and stabilized using a thermal blanket. A filament was carefully inserted into the external carotid artery and meticulously advanced toward the internal carotid artery, ultimately reaching the middle cerebral artery. For screening stroke-sensitive and stroke-resistant mice, focal cerebral ischemia was induced by 90 min MCAO (*n* = 36). After cerebral ischemia, the filament was withdrawn to facilitate the commencement of reperfusion. Animals were excluded if they died during MCAO surgery or showed no evidence of induced cerebral infarction, as assessed by T2-weighted magnetic resonance imaging (MRI) or 2,3,5-triphenyltetrazolium chloride (TTC) staining (i.e., no detectable infarct). Survival rates of mice were assessed up to day 7 after MCAO. Mice reaching humane endpoints within day 1 after MCAO were defined as ischemic stroke-sensitive (SEN; *n* = 13), while those that survived on day 7 after MCAO were characterized as ischemic stroke-resistant (RES; *n* = 10). After 7 days of treatment, all mice were humanely euthanized. Animals were continuously monitored during day 1 (the first 24 h) after MCAO (including overnight) by trained personnel on a rotating schedule. Animals reaching predefined humane endpoints (e.g., more than 20% baseline body weight, complete paralysis without spontaneous movement, severe ataxia/loss of postural reflexes, or severe deterioration with poor grooming), consistent with the previous study, were immediately anesthetized, euthanized, and sampled (25). Blood was collected via the abdominal aorta and processed for serum, and tissues were harvested immediately using an identical protocol for daytime and nighttime. Mice were obtained as littermate cohorts, and litter information was recorded. SEN and RES phenotypes were distributed across the same litters without evidence of a single-litter bias.

### Fecal microbiota transplantation experiment

Fecal microbiota transplantation (FMT) was performed according to the previously described method (26). In brief, the microbiota donors were SEN and RES mice. Preoperatively collected feces of SEN and RES mice were stored at −80°C for further experiments. As microbiota recipients, pseudo-germ-free mice (8-week-old male C57BL/6J) were established by administering broad-spectrum antibiotics (ABX) for 1 week to deplete the gut microbiota (vancomycin: 100 mg/kg/day; neomycin sulfate: 200 mg/kg/day; metronidazole: 200 mg/kg/day; ampicillin: 200 mg/kg/day). Donor fecal samples were collected preoperatively and prepared using donors from multiple litters within each phenotype group to reduce potential litter-related confounding. The SEN_recipient group (SEN_R, *n* = 7) received pooled donor stool from SEN mice (SEN, *n* = 13), whereas the RES_recipient group (RES_R, *n* = 7) received pooled donor stool from RES mice (RES, *n* = 10). Fecal suspensions were prepared by thoroughly vortexing 1.25 g of donor feces in 10 mL sterile phosphate-buffered saline, followed by centrifugation at 500 × *g* for 1 min to pellet insoluble debris. Each recipient mouse was administered 0.15 mL of the suspension by oral gavage once daily for 1 week.

### Microbial profiling

Cecal contents were collected, snap-frozen in liquid nitrogen, and stored at −80°C until processing by microbiomics. Bacterial DNA was extracted from samples of colonic contents using the QIAamp Fast DNA Stool Mini Kit (QIAGEN, Valencia, CA, USA) according to the manufacturer’s protocols. The V4 hypervariable region of the bacterial 16S rRNA gene was amplified using *16S-V4* barcoded primers (Table S1). The amplified products were sequenced on the Illumina MiSeq platform (2 × 250 bp paired-end sequencing). The raw sequences were processed via the DADA2 pipeline to obtain amplicon sequence variants (ASVs). Subsequently, ASVs were annotated taxonomically using the Greengenes database (v13.8) with a threshold of 97% similarity. The taxonomic data were used for further analysis. The alpha and beta diversity were conducted in the R package microeco (v1.4.0). Alpha diversity was estimated using the Chao1 and Shannon indices. Beta diversity was computed using the Jaccard distance and visualized through principal coordinates analysis (PCoA). The significance of community structure differences among groups was determined using permutational multivariate analysis of variance (PERMANOVA), implemented through the adonis2 function of the vegan package in R, based on 999 permutations. Differential taxa between groups across all taxonomic levels were identified using linear discriminant analysis (LDA) effect size (LEfSe). *P*-values were corrected for multiple comparisons using the Benjamini-Hochberg false discovery rate (FDR) method, and taxa with an FDR-adjusted *P* < 0.05 and an LDA score > 2.0 were retained.

### Untargeted metabolomic profiling

Cecal contents were collected, snap-frozen in liquid nitrogen, and stored at −80°C until untargeted metabolomic analysis. The cecal contents were placed in microcentrifuge tubes and resuspended in prechilled 80% methanol by vigorous vortexing and then thawed on ice and vortexed for 30 s. Following 6 min of sonication, the samples were centrifuged at 5,000 rpm at 4°C for 1 min. The supernatant was lyophilized and dissolved in 10% methanol. Finally, the solution was injected into the liquid chromatography-mass spectrometry/mass spectrometry (LC-MS/MS) for analysis. LC-MS/MS-based untargeted metabolomic profiling was performed using a Vanquish UHPLC system (Thermo Fisher) coupled with an Orbitrap Q Exactive HF mass spectrometer (Thermo Fisher) in Novogene Co., Ltd. (Beijing, China). The raw data files generated by LC-MS/MS were processed using Compound Discoverer 3.3 (CD3.3, Thermo Fisher) for peak alignment, picking, and metabolite quantitation. The normalized data were used to predict molecular formulas based on additive ions, molecular ion peaks, and fragment ions. Peaks were aligned using the mzCloud, mzVault, and MassList databases to obtain accurate qualitative and relative quantitative results.

Metabolites were annotated employing the Kyoto Encyclopedia of Genes and Genomes (KEGG) database. The statistical evaluation of untargeted metabolomic data was performed using multidimensional statistical methods, such as principal component analysis (PCA) and partial least squares discriminant analysis (PLS-DA). PLS-DA model was constructed on mean-centered and scaled metabolite data. Model performance was evaluated by sevenfold cross-validation, and goodness of fit and predictive ability were summarized using *R*^2^ and *Q*^2^, respectively. Potential overfitting was assessed by permutation testing (200 random permutations of the class labels). For each permutation, a PLS-DA model was built, and *R*^2^/*Q*^2^ were recorded to generate regression lines against the correlation between original and permuted class labels. Models were considered valid when the original *R*^2^ exceeded *Q*^2^ and the *Q*^2^ regression intercept was <0.

Differential metabolite analysis was performed as follows. After data preprocessing and normalization, the relative abundance values of metabolites were log_2_-transformed prior to statistical analysis to approximate normal distribution. Each metabolite was individually compared between groups using two-sided Welch’s *t*-test, and the resulting *P*-values were adjusted for multiple comparisons using the Benjamini-Hochberg FDR method. Metabolites with FDR-adjusted *P* < 0.05 and fold change (FC) > 1.5 or FC < 0.667 were considered statistically significant.

### Integration of microbiome and metabolome associations

For microbiome-metabolome integration, microbiome profiles were summarized at the genus level and expressed as relative abundances. Metabolite intensities were represented by the integrated chromatographic peak areas of each feature and normalized to the total peak area within each sample. Integration analyses were restricted to the set of differentially abundant genera and the key differential metabolites. Feature-level associations between genera and metabolites across all samples were evaluated using Spearman’s correlation. For each genus-metabolite pair, Spearman’s correlation coefficient (Spearman’s *r*) and the corresponding *P* value were calculated, and multiple comparisons were controlled using the Benjamini-Hochberg FDR-adjusted *P*-values.

### Histopathological analysis

Brain tissues were harvested and sectioned, followed by immersion staining of unfixed brain slices with 1% TTC staining to determine brain infarction. The infarct area of the image was analyzed using Image-Pro Plus 6.0 software (Media Cybernetics, Inc.), excluding the influence of confounding factors such as cerebral edema during the analysis. The percentage of brain infarct area relative to the entire hemisphere was calculated as follows: brain infarction (%) = (the sum of infarct areas across all infarcted slices)/(contralateral cerebral area × 2) × 100%.

Liver, lung, kidney, and ileum tissues were removed from the mice, fixed with 4% paraformaldehyde, and embedded in paraffin according to standard procedures. Tissues embedded in paraffin were sectioned and further subjected to hematoxylin-eosin (H&E) staining. Images were acquired using a digital slide scanner and slide viewer software (Pannoramic MIDI and Case Viewer, 3D Histech, Hungary).

### Magnetic resonance imaging and image processing

Ten male C57BL/6J mice were subjected to 90 min MCAO. Four mice that met the humane endpoints within day 1 were classified as SEN (*n* = 4), whereas five mice survived to day 7 and were classified as RES (*n* = 5). T2-weighted MRI was performed to quantify infarct volume after MCAO, with imaging conducted in SEN mice at day 1 and in RES mice at days 1, 2, and 7. MRI acquisition was conducted on a 7.0 Tesla small animal magnetic resonance scanner (PharmaScan 70/16 US, Burker Biospin MRI GmbH) running Paravision 6.0 software as previously described (27). In brief, mice were anesthetized with 1.5% isoflurane and positioned in a cradle containing a heating pad to maintain body temperature and placed in the MRI scanner. Respiration and heart rate were continuously monitored through a respiratory/electrocardiogram unit. T2-weighted imaging was acquired using a 2D fast-spin echo sequence with the following parameters: repetition time 3,000 ms, echo time 36 ms, field of view 25.6 × 25.6 mm, matrix of 256 × 256, slice thickness 0.7 mm. DICOM data were exported and analyzed using RadiAnt DICOM Viewer. Infarct regions were manually delineated on T2-weighted images slice-by-slice by two independent trained raters blinded to group assignment. Infarct volume was calculated by summing infarct areas across slices and multiplying by slice thickness and is reported as a percentage of total brain volume. Infarct size was expressed as a percentage of the whole brain using the following formula: brain infarction (%) = (sum of the infarct volume)/(contralateral cerebral volume × 2) × 100%.

### Biochemical analysis

Serum interleukin-6 (IL-6), interleukin-1 beta (IL-1β), and lipopolysaccharide (LPS) were measured using the enzyme-linked immunosorbent assay kits (Meimian, China) according to the manufacturer’s instructions. Alanine aminotransferase (ALT) and aspartate aminotransferase (AST) levels in the serum were determined using the respective ALT and AST assay kits (Nanjing Jiancheng, China) in accordance with the manufacturer’s protocols.

### Quantitative real-time polymerase chain reaction

RNA was extracted from tissue using TRIzol reagent (Accurate Biology) in combination with the bead beating and phenol:chloroform:isoamyl alcohol method as previously described (17). RNA was reverse-transcribed using the Evo M-MLV RT premix kit (Accurate Biology), and the resulting cDNA was used as the template for quantitative real-time polymerase chain reaction (qPCR). qPCR was performed using an AceQ qPCR SYBR Green Master Mix (Vazyme). The specific sequences of the primers used for the detection of each gene were summarized separately in Table S1. The relative expression of each primer between the two groups was determined using the 2^−ΔΔCt^ method, with the values normalized to the reference housekeeping gene *Gapdh*.

### Statistical analysis

Data were expressed as mean ± standard error of the mean (mean ± SEM). The differences between two groups were assessed using the two-sided Student’s *t*-test for normally distributed data, or Wilcoxon rank-sum test for non-normally distributed variables. Comparisons among more than two groups were performed using one-way analysis of variance followed by Tukey’s test, unless otherwise explained. Where applicable, multiple testing was controlled using the Benjamini-Hochberg FDR correction method. Statistical analyses were performed with GraphPad Prism 9 (GraphPad Software Inc.; San Diego, CA, USA) and R software version 4.3.3. Differences of *P* < 0.05 were considered significant in all statistical analyses.

## RESULTS

### Characterization of MCAO-sensitive and MCAO-resistant mice

**Fig 1:**
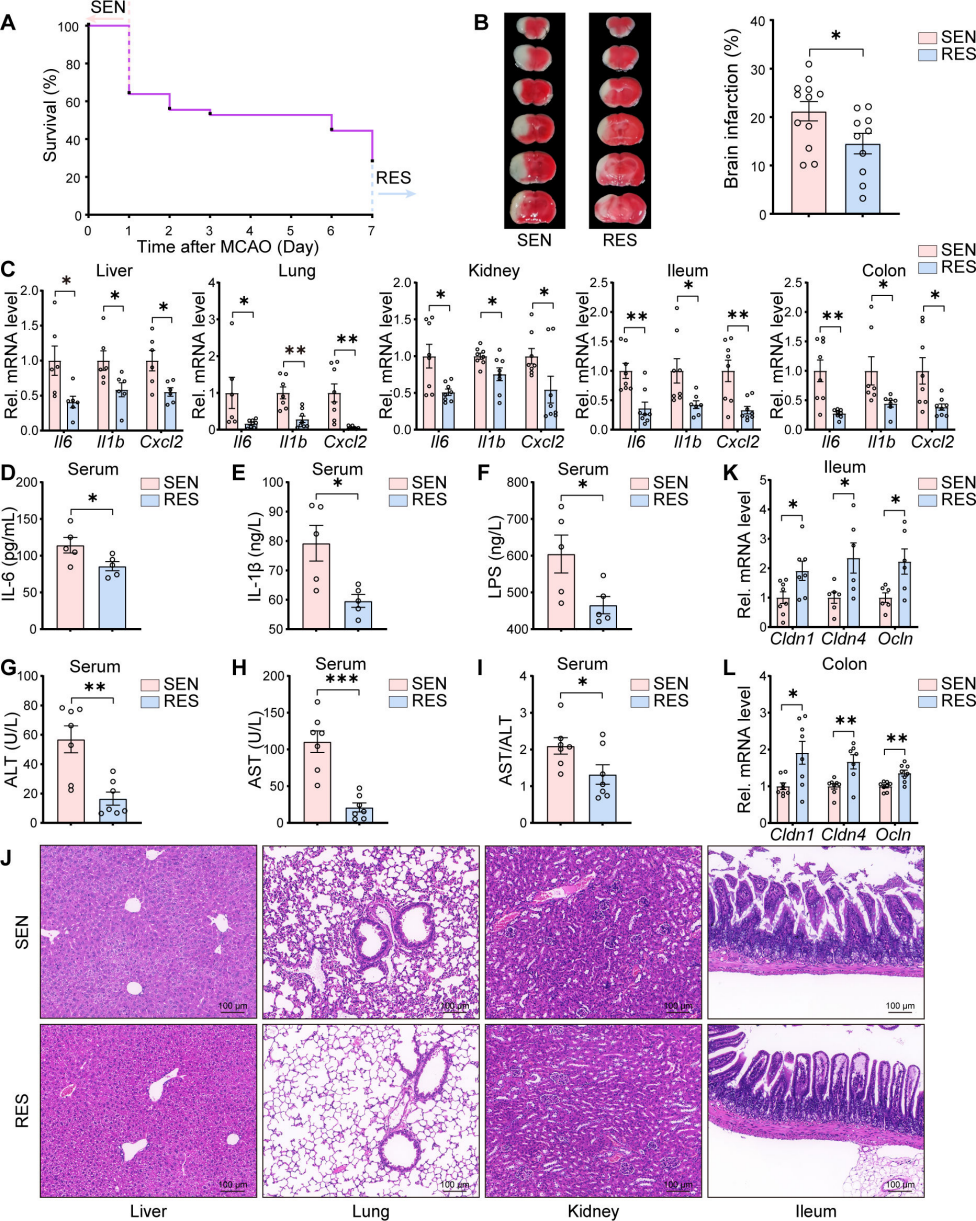
Characterization of SEN and RES mice. (**A**) 90 min MCAO was subjected to screen mice based on their survival time after ischemic stroke. SEN (*n* = 13): reaching humane endpoints within day 1. RES (*n* = 10): surviving to day 7. (**B**) The representative of brain sections according to TTC staining and percentage of brain infarction area (*n* = 10–12 per group). (**C**) mRNA expression levels of pro-inflammatory cytokines (*Il6*, *IL1b*, and *Cxcl2*) in liver, lung, kidney, ileum, and colon tissues (*n* = 6–8 per group). Serum (**D**) IL-6, (**E**) IL-1β, (**F**) LPS level (*n* = 5 per group). Serum (**G**) ALT and (**H**) AST levels and (**I**) the AST/ALT ratio (*n* = 7 per group). (**J**) H&E-stained histopathological sections of liver, lung, kidney, and ileum tissues. Scale bar = 100 µm. mRNA expression levels of tight junction-related genes (*Cldn1*, *Cldn4*, and *Ocln*) in (**K**) ileum and (**L**) colon tissues (*n* = 6–8 per group). Data were expressed as mean ± SEM. Statistical analysis was assessed using the two-sided Student’s *t*-test or the Wilcoxon rank-sum test. *P < 0.05, **P < 0.01, ***P < 0.001. ALT, alanine aminotransferase; AST, aspartate aminotransferase; *Cldn1*, claudin-1; *Cldn4*, claudin-4; *Cxcl2*, C-X-C motif chemokine ligand 2; H&E, hematoxylin-eosin; *Il1b*, interleukin 1 beta; IL-1β, interleukin-1 beta; *Il6*, interleukin 6; IL-6, interleukin-6; LPS, lipopolysaccharide; MCAO, middle cerebral artery occlusion; *Ocln*, occludin; RES, ischemic stroke-resistant; SEN, ischemic stroke-sensitive; TTC, 2,3,5-triphenyltetrazolium chloride.

Mice were subjected to MCAO for 90 min to mimic stroke injury. Mice that met humane endpoints within day 1 after MCAO were classified as ischemic stroke-sensitive (SEN), whereas mice that survived to post-MCAO day 7 were classified as ischemic stroke-resistant (RES) (Fig. 1A). Tissues from SEN mice that reach humane endpoints were harvested immediately, and RES mice were euthanized and sampled on day 7. Compared with SEN mice, RES mice exhibited a significantly smaller infarct volume by TTC staining (Fig. 1B) and significantly lower mRNA levels of proinflammatory cytokines, including interleukin 6 (*Il6*), interleukin one beta (*Il1b*), and C-X-C motif chemokine ligand 2 (*Cxcl2*), across multiple organs (liver, lung, kidney, ileum, and colon) (Fig. 1C). Moreover, the serum levels of IL-6, IL-1β, and LPS were significantly lower in RES mice than those in SEN mice (Fig. 1D through F). Of note, liver injury was mitigated in RES mice, as demonstrated by reduced levels of ALT, AST, and as well as the AST/ALT ratio in RES mice (Fig. 1G through I). Representative H&E-stained sections of the liver, lung, kidney, and ileum showed milder histopathological abnormalities in RES than SEN mice (Fig. 1J). Compared with SEN mice, RES mice showed marked alleviation of intestinal barrier impairment, which was associated with significantly increased mRNA expression levels of the tight junction-related genes claudin-1 (*Cldn1*), claudin-4 (*Cldn4*), and occludin (*Ocln*) in both the ileum and colon (Fig. 1K and L). These results collectively indicate that SEN mice exhibit more severe brain injury than RES mice, accompanied by increased systemic inflammation and elevated intestinal permeability.

To characterize the dynamic progression of brain injury after stroke, we performed longitudinal T2-weighted MRI to quantify ischemic lesion volumes (Fig. S1A). In RES mice, infarct volumes measured on post-stroke days 1, 2, and 7 were comparable, with no significant differences observed across time points (Fig. S1B), indicating that the infarct burden was largely established early after MCAO and remained stable thereafter, with minimal delayed expansion. To directly compare early injury severity, T2-weighted MRI was additionally performed on day 1 in all mice while they were still alive. At this early time point, SEN mice exhibited significantly larger infarct volumes than RES mice (Fig. S1C), demonstrating that infarct severity diverged early after stroke onset between SEN and RES mice. Collectively, these findings indicate substantial differences in susceptibility to ischemic brain injury between SEN and RES mice.

### Susceptibility of ischemic stroke-induced organ injury was transmissible by gut microbiota

**Fig 2:**
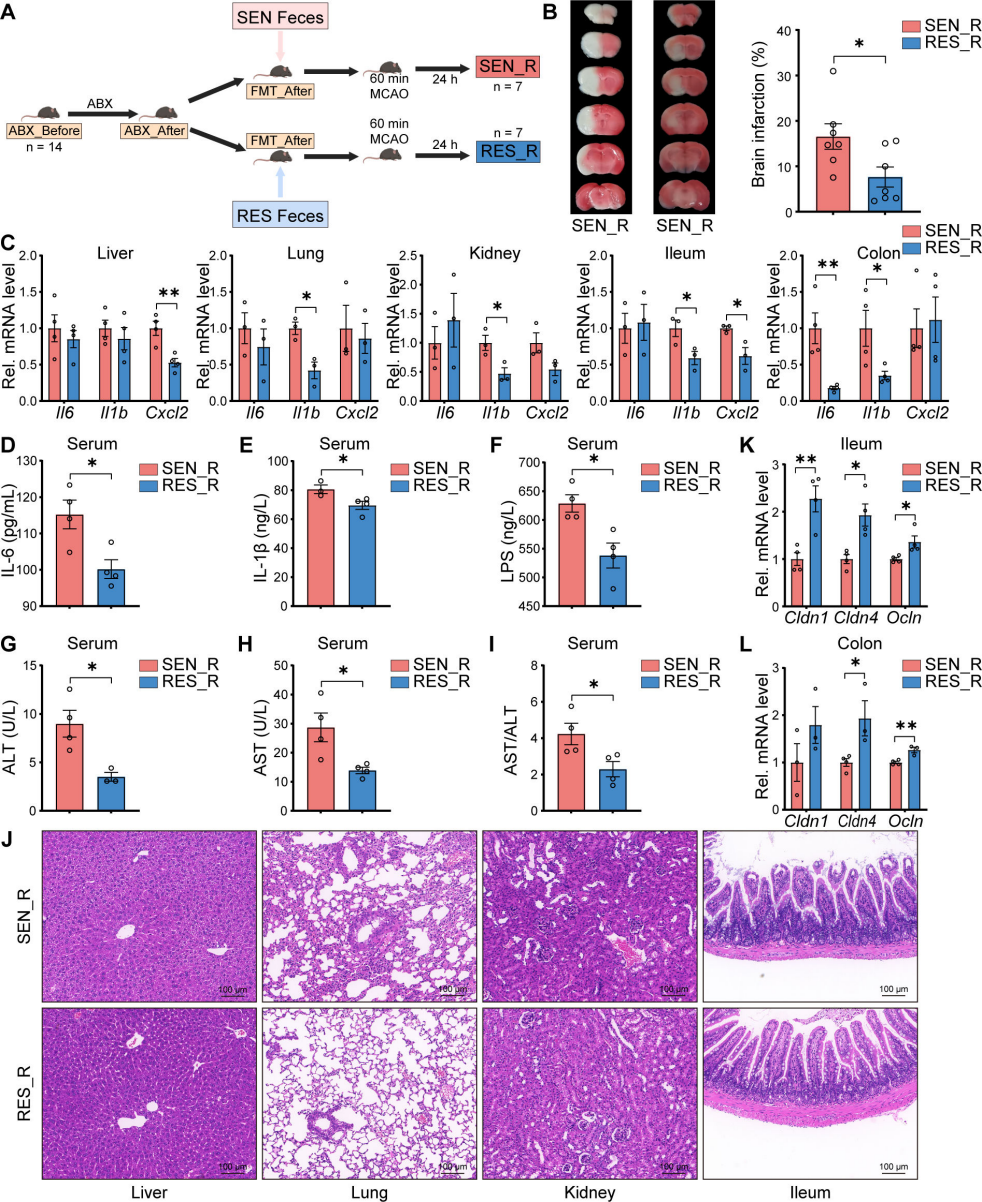
Vulnerability of ischemic stroke-induced tissue injury was transmissible by gut microbiota. (**A**) Schematic diagram of the FMT experiment. Created with BioRender.com. (**B**) The representative of brain sections according to TTC staining and percentage of brain infarction area in the recipient mice (*n* = 7 per group). (**C**) mRNA expression levels of pro-inflammatory cytokines (*Il6*, *Il1b*, and *Cxcl2*) in liver, lung, kidney, ileum, and colon tissues in the recipient mice (*n* = 3– 4per group). Serum (**D**) IL-6, (**E**) IL-1β, and (**F**) LPS level in the recipient mice (*n* = 3–4 per group). Serum (**G**) ALT, (**H**) AST, and (**I**) the AST/ALT ratio in the recipient mice (*n* = 3–4 per group). (**J**) H&E-stained histopathological sections of liver, lung, kidney, and ileum tissues in the recipient mice. Scale bar = 100 µm. mRNA expression levels of tight junction-related genes (*Cldn1*, *Cldn4*, and *Ocln*) in (**K**) ileum and (**L**) colon tissues in the recipient mice (*n* = 3–4 per group). Data were expressed as mean ± SEM. Statistical analysis was assessed using the two-sided Student’s *t*-test or the Wilcoxon rank-sum test. *P < 0.05, **P < 0.01. ALT, alanine aminotransferase; AST, aspartate aminotransferase; *Cldn1*, claudin-1; *Cldn4*, claudin-4; *Cxcl2*, C-X-C motif chemokine ligand 2; FMT, fecal microbiota transplantation; H&E, hematoxylin-eosin; *Il1b*, interleukin 1 beta; IL-1β, interleukin-1 beta; *Il6*, interleukin 6; IL-6, interleukin-6; LPS, lipopolysaccharide; MCAO, middle cerebral artery occlusion; *Ocln*, occludin; RES_R, RES_recipient; SEN_R, SEN_recipient; TTC, 2,3,5-triphenyltetrazolium chloride.

Next, our study explored the potential factors mediating the differences in susceptibility to MCAO. Previous research indicated that changes in gut permeability in mice were closely related to the composition of the gut microbiota (28). We attempted to investigate whether the different susceptibilities to stroke injury between SEN and RES mice resulted from the gut microbiota. The FMT experiment was performed to confirm the direct role of altered gut microbiota by ischemic stroke. Mice were treated with ABX for 1 week to eliminate the gut microbiota, followed by transplantation of gut microbiota from SEN or RES mice for 1 week, after which the recipient mice were subjected to 60 min MCAO and sacrificed at 24 h after stroke (Fig. 2A). To confirm bacterial depletion and recolonization, qPCR was performed to measure 16S rRNA bacterial load in fecal sample. The results showed a marked reduction in bacterial load after antibiotic treatment, demonstrating effective gut microbiota depletion. After FMT, the bacterial load recovered to baseline levels, indicating successful recolonization (Fig. S2). Mice receiving microbiota from SEN mice were defined as SEN_R, and mice receiving microbiota from RES mice were defined as RES_R. As a result, the infarct volume was significantly smaller in RES_R mice than in SEN_R mice based on TTC staining (Fig. 2B). In addition, the mRNA level of *Il6* in the colon; levels of *Il1b* in the lung, kidney, ileum, and colon; and levels of *Cxcl2* in the liver and ileum were lower in RES_R mice than those in SEN_R mice (Fig. 2C). As expected, serum levels of IL-6, IL-1β, and LPS were lower (Fig. 2D through F), with reduced levels of ALT, AST, and the AST/ALT ratio in RES_R mice than those in SEN_R mice (Fig. 2G through I). Histopathological changes across multiple organs (liver, lung, kidney, and ileum) were also alleviated in RES_R mice (Fig. 2J), with increased levels of mRNA expressions of tight junction-related genes in the ileum and colon (Fig. 2K and L). Taken together, these findings demonstrate that the gut microbiota mediates the susceptibility of stroke injury in mice.

### The difference in gut microbiota between SEN and RES mice

**Fig 3:**
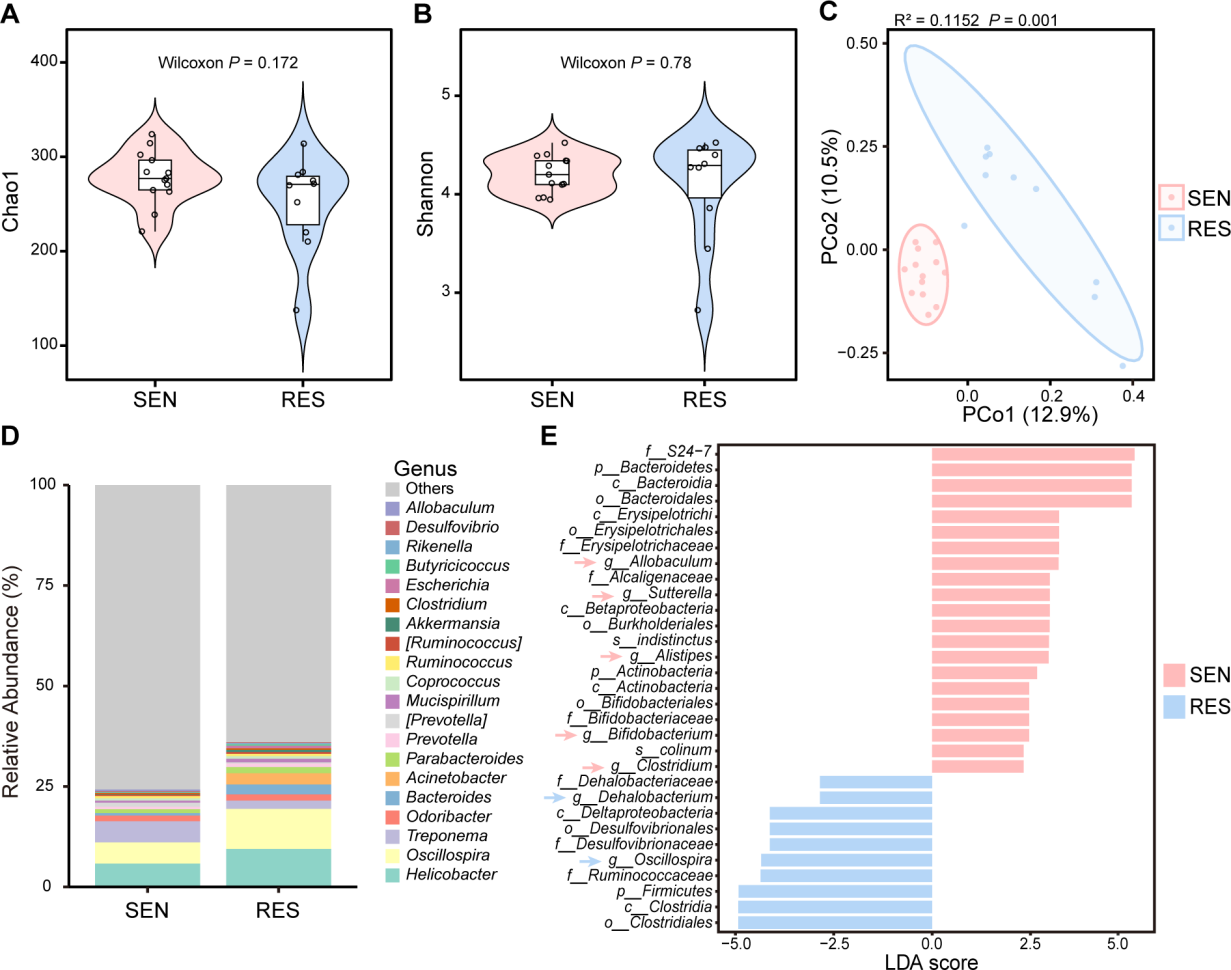
Gut microbiota from SEN and RES exhibited different compositions. Comparison of alpha diversity indices (**A**) Chao1 index, (**B**) Shannon index between groups (*n* = 10–13 per group). Statistical analysis was assessed using the Wilcoxon rank-sum test. (**C**) PCoA based on Jaccard distances showed clear separation between SEN and RES samples (*n* = 10–13 per group). Statistical significance was assessed using Adonis/PERMANOVA. Solid ellipse indicated the 95% confidence intervals. (**D**) The composition of the gut microbiota at the genus level. (**E**) Differential taxa between groups across all taxonomic levels were determined by LEfSe with Benjamini-Hochberg FDR method (only those taxa with FDR-adjusted *P* < 0.05 and LDA > 2.0 were shown). The letters in front of the bacteria represented classifying organism markers: *p*, phylum; *c*, class; *o*, order; *f*, family; *g*, genus. The light pink arrow indicated the genus significantly enriched in the SEN group, whereas the light blue arrow indicated the genus significantly enriched in the RES group. FDR, false discovery rate; LEfSe, linear discriminant analysis effect size; LDA, linear discriminant analysis; PCoA, principal coordinate analysis; RES, ischemic stroke resistant; SEN, ischemic stroke sensitive.

Next, we investigated the gut microbiota composition in SEN and RES mice using 16S rRNA gene sequencing of cecal contents. Alpha-diversity indices, such as Chao1 index and Shannon index, did not differ between the two groups (Fig. 3A and B). Nevertheless, beta-diversity based on Jaccard distance was significantly different between the two groups, as manifested by a clear separation of two groups in PCoA (Fig. 3C). At the genus level, RES mice showed a higher relative abundance of *Oscillospira* and *Helicobacter* and a lower relative abundance of *Treponema* than SEN mice (Fig. 3D). LEfSe analysis identified differential microbial taxa between the two groups, with the genera of *Allobaculum*, *Sutterella*, *Alistipes*, *Bifidobacterium*, and *Clostridium* enriched in the SEN group, whereas the genera of *Oscillospira* and *Dehalobacterium* were increased in the RES group (Fig. 3E; Table S2). Particularly, we observed substantially elevated levels of butyrate-producing bacteria in RES mice, including *Clostridiales* (29, 30), *Clostridia* (31, 32), *Ruminococcaceae* (33), and *Oscillospira* (34). Collectively, our results show that there are significant differences in the composition of the gut microbiota in cecal contents between SEN and RES mice.

### Untargeted metabolomics to identify pivotal metabolites

**Fig 4:**
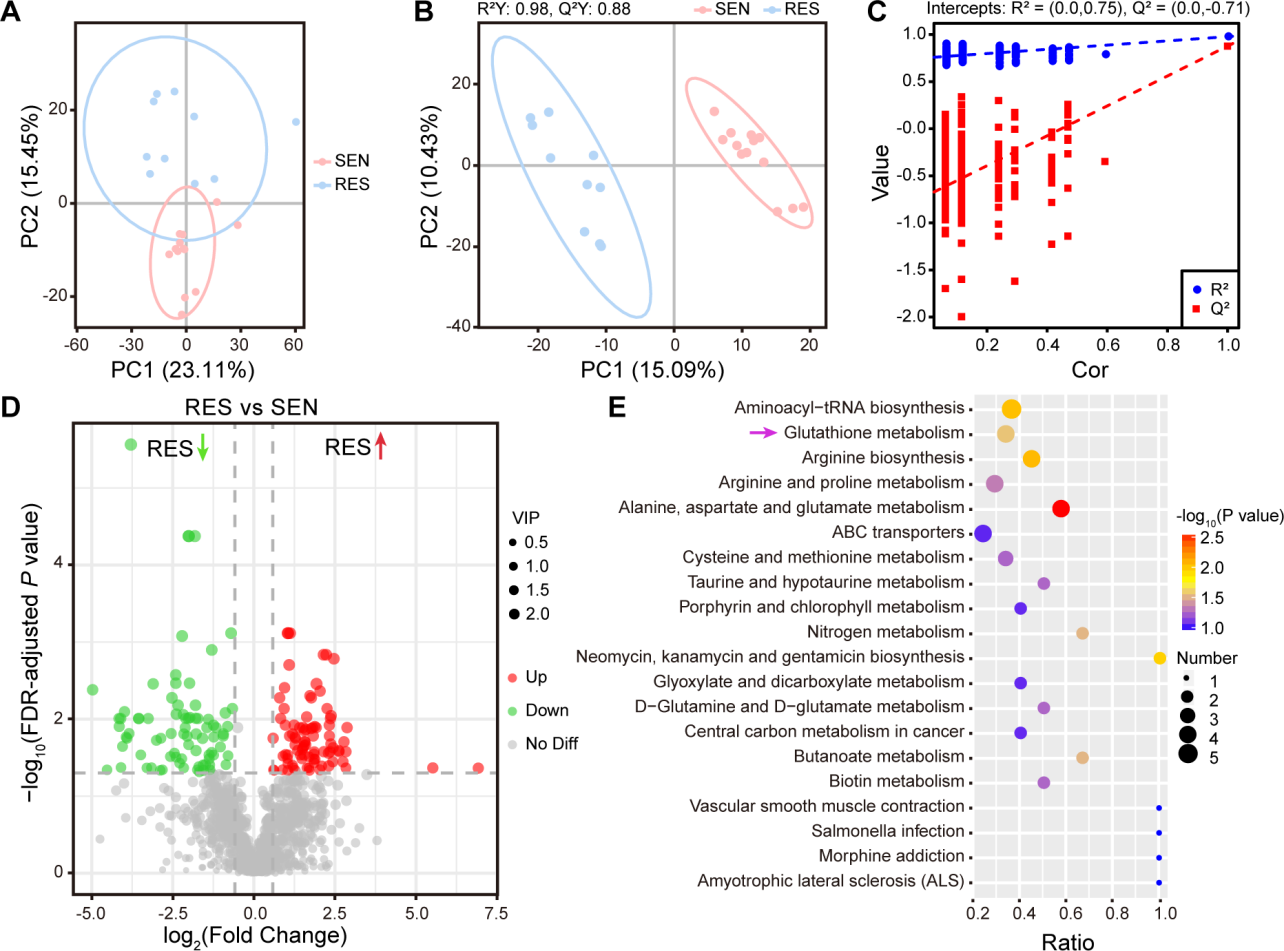
Differences in the gut microbiota-derived metabolites between ischemic SEN and RES mice after MCAO induction. (**A**) PCA score plot of metabolomic profiles from SEN and RES mice (*n* = 10–13 per group). Ellipse indicated the 95% confidence intervals. (**B**) PLS-DA of metabolites between the SEN and RES groups (*n* = 10–13 per group). Ellipse indicated the 95% confidence intervals. (**C**) Cross-validation model of PLS-DA. (**D**) Differential metabolites between the RES and SEN groups (*n* = 10–13 per group). Volcano plot showing 168 significantly altered metabolites in RES versus SEN groups, including 87 upregulated and 81 downregulated metabolites. Differential metabolites were defined by FDR-adjusted *P* value < 0.05 and FC > 1.5 or FC < 0.667. (**E**) The top 20 enriched KEGG pathways of differential metabolites. KEGG pathway enrichment (hypergeometric test) performed on the combined list of both upregulated and downregulated differential metabolites against the background of all annotated metabolites. The *x*-axis indicated the enrichment ratio. The *y*-axis represented KEGG pathway. Bubble size represented the number of differential metabolites in the pathway, and bubble color indicated statistical significance. The purple arrow indicated the glutathione metabolism pathway, which was prominently enriched among the differential metabolites. FC, fold change; FDR, false discovery rate; MCAO, middle cerebral artery occlusion; PCA, principal component analysis; PLS-DA, partial least squares-discriminant analysis; RES, ischemic stroke resistant; SEN, ischemic stroke sensitive; VIP, variable importance in the projection.

To investigate the differential metabolites between SEN and RES mice, cecal contents were analyzed using metabolomics on the LC-MS/MS. Multivariate PCA, an unsupervised statistical method suitable for analyzing and classifying metabolomic data sets, was employed to identify intrinsic clusters of metabolomic profiles. The first principal component (PC1) accounted for 23.11% of the variance, PC2 accounted for 15.45%, respectively (Fig. 4A). Additionally, PLS-DA, a supervised discriminant analysis statistical method, was utilized to detect the differences within and between SEN and RES metabolite profile changes in cecal contents. The R^2^Y and Q^2^Y values of the PLS-DA model, which were 0.98 and 0.88, respectively, indicated excellent model fit and predictive ability. According to PLS-DA, samples within the same group clustered together, while different groups were distinctly separated, demonstrating variations between SEN and RES groups (Fig. 4B). To test the validity of the models of PLS-DA, a permutation test (*n* = 200) was conducted, confirming its reliability in identifying differential metabolites (Fig. 4C). Metabolites with significant differences between the RES and SEN groups were identified based on FDR-adjusted *P* < 0.05 and FC > 1.5 or FC < 0.667. A total of 168 differential metabolites were obtained from the volcano plot, of which 87 were significantly upregulated and 81 were downregulated in the RES group compared to the SEN group (Fig. 4D; Table S3). Moreover, differential metabolites were annotated using the KEGG database, and the top 20 enriched KEGG pathways based on these differential metabolites were shown in Fig. 4E. Particularly, it revealed a significant enrichment of metabolites involved in glutathione metabolism. These findings underscore substantial metabolic alterations in the cecal contents of mice sensitive and tolerant to ischemic stroke.

### Integrated analysis of gut microbiome and metabolome

**Fig 5:**
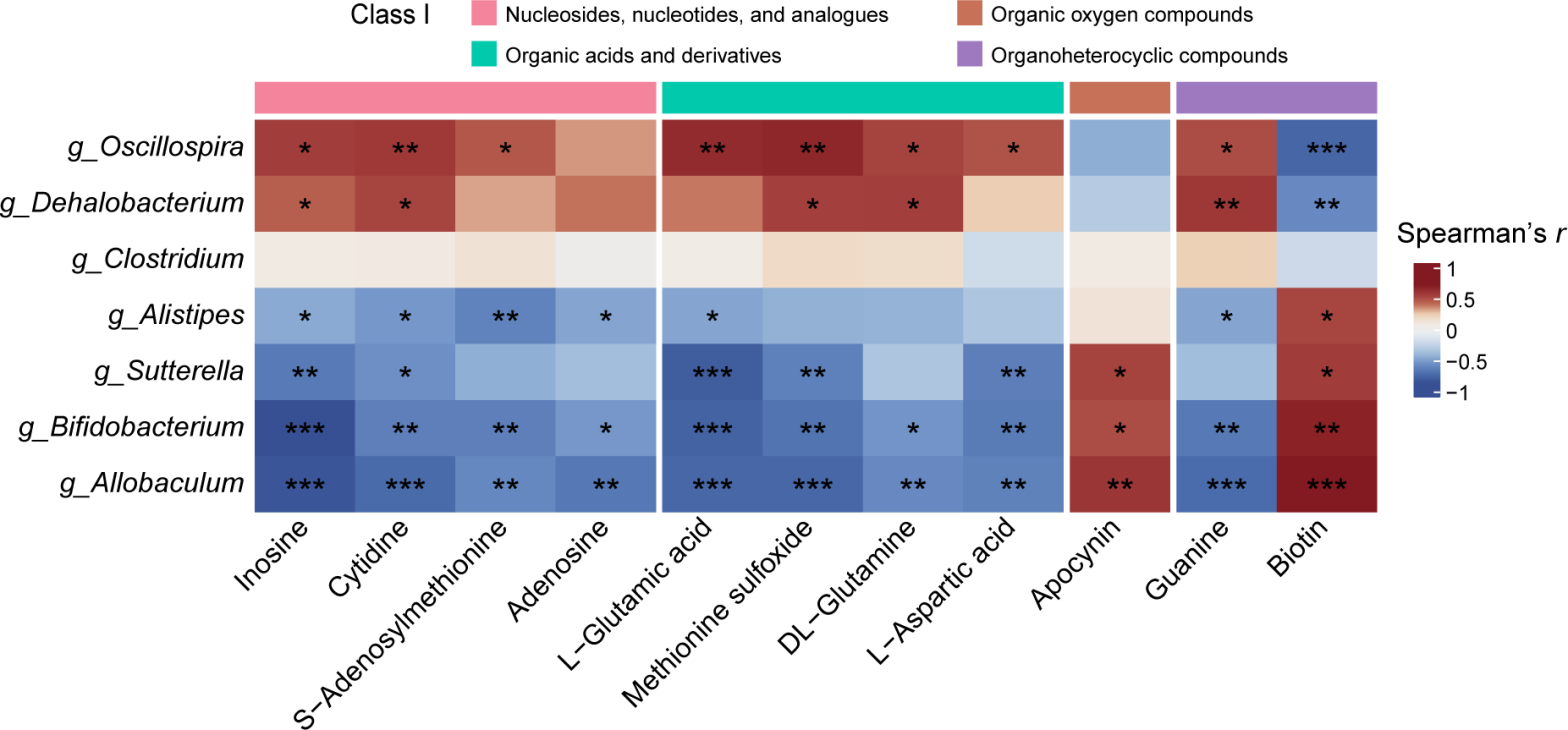
Correlation analysis between differential gut microbiota and metabolites in cecal contents. Spearman’s correlation analysis between the 7 different genera and the 11 key differential metabolites (inosine, cytidine, S-adenosylmethionine, adenosine, L-glutamic acid, methionine sulfoxide, DL-glutamine, L-aspartic acid, apocynin, guanine, and biotin). Red and blue denoted positive and negative correlations, respectively, with color intensity proportional to the correlation coefficient (Spearman’s *r*). *P*-values were adjusted for multiple testing using the Benjamini-Hochberg FDR method. Significant correlations are marked with asterisks. FDR-adjusted *P* < 0.05 (*), FDR-adjusted *P* < 0.01 (**), FDR-adjusted *P* < 0.001(***). FDR, false discovery rate.

To investigate whether changes in gut microbiota were related to alterations in intestinal metabolites and to elucidate the relationship between key differential gut microbes and metabolites, integrated analysis of 16S rRNA gene sequencing and untargeted metabolomics by LC-MS/MS of paired cecal contents was applied to identify gut microbiome-associated metabolites with significantly altered abundance in SEN and RES mice. We conducted a Spearman’s correlation analysis to detect the association between gut microbiome and metabolome. Our study linked 7 different genera (*Oscillospira*, *Dehalobacterium*, *Clostridium*, *Alistipes*, *Sutterella*, *Bifidobacterium*, and *Allobaculum*) (Fig. 3E) of the SEN mice and RES mice with 11 key differential metabolites (inosine, cytidine, S-adenosylmethionine, adenosine, L-glutamic acid, methionine sulfoxide, DL-glutamine, L-aspartic acid, apocynin, guanine, and biotin) that were fully matched in mzCloud, mzVault, and Masslist databases (Table S3), revealing correlations between certain differential microbes and metabolites (Fig. 5A). For instance, Spearman’s correlation analysis showed that biotin was positively correlated with *Allobaculum* (FDR-adjusted *P-*value < 0.001), *Bifidobacterium* (FDR-adjusted *P-*value < 0.01), *Sutterella* (FDR-adjusted *P*-value < 0.05)*,* and *Alistipes* (FDR-adjusted *P*-value < 0.05), and negatively correlated with *Oscillospira* (FDR-adjusted *P*-value < 0.001) and *Dehalobacterium* (FDR-adjusted *P*-value < 0.01). These findings indicate that gut microbiota-derived metabolic alterations may contribute to the differential phenotypes observed between SEN and RES mice.

## DISCUSSION

Our study demonstrates that predisposition to severe ischemic injury is transmissible through the gut microbiota. By stratifying mice into SEN and RES phenotypes and performing preoperative FMT, we provide functional evidence supporting a causal role of the gut microbiota in determining stroke severity. Integrative 16S rRNA gene sequencing combined with LC-MS/MS-based untargeted metabolomics further identified candidate microbial taxa and metabolic pathways, including butyrate-associated bacteria and glutathione-related metabolites, which may underlie this differential vulnerability. Collectively, these findings link gut microbial dysbiosis and metabolic perturbations with susceptibility to ischemic injury and suggest that microbiota-targeted interventions may represent a potential strategy for reducing stroke risk.

Accumulating evidence supports a bidirectional gut-brain axis in ischemic stroke, in which the gut microbiota can influence brain injury and systemic inflammation, while stroke itself can rapidly disrupt intestinal homeostasis. In experimental models, antibiotic-driven perturbation of the intestinal microbiota reduces ischemic brain injury, and this effect is transmissible by FMT, establishing a causal role of the microbiota in shaping stroke outcomes (35). Consistent with this concept, ischemic stroke has been shown to trigger rapid gut dysbiosis that can exacerbate infarction and inflammation (36), and clinical-translational studies further indicate that gut dysbiosis signatures correlate with stroke severity and outcome, with patient-to-mouse FMT providing causal support (37). Beyond stroke *per se*, comorbidity-associated microbiota may also aggravate ischemic injury. For example, type 2 diabetes-associated microbiota exacerbates stroke outcomes in a manner supported by FMT-based causal testing (23). Recent work additionally highlights neurogenic pathways linking stroke-related stress to gut barrier dysfunction and altered host-microbe interactions (38). Despite these advances, it remains unclear whether baseline differences in the gut microbiota can determine intrinsic susceptibility and vulnerability to severe ischemic injury among hosts with littermates of the same genetic background exposed to an identical ischemic insult. Here, we established a “susceptibility-first” framework by stratifying mice into SEN and RES phenotypes based on survival following standardized MCAO, and by transplanting preoperatively collected feces from SEN or RES donors into antibiotic-treated recipients prior to MCAO. This design directly tests whether predisposition to severe ischemic injury is transmissible via the gut microbiota. In addition, our findings highlight candidate microbial lineages and metabolic pathways, such as butyrate-associated taxa and metabolites related to glutathione metabolism that may contribute to differential susceptibility and provide mechanistic hypotheses for future validation.

Despite the widespread use to enhance experimental reproducibility, substantial phenotypic heterogeneity can still arise within the commonly employed C57BL/6 genetic background. Prior studies have shown that C57BL/6 substrains can exhibit distinct disease-related phenotypes and differential susceptibility under pathological challenges, including insulin responses to high-fat feeding (39), substrain-specific congenital coronary anatomy shaping cardiovascular vulnerability (40), and heterogeneous cardiac function in response to pressure overload (41). Importantly, both substrain and biological sex are recognized modifiers of ischemic stroke phenotypes, suggesting that substrain and sex differences affect stroke vulnerability (42, 43). Phenotypic dispersion is also observed among littermates in other contexts, underscoring that meaningful inter-individual variability can occur even within a shared genetic background (44). In line with these observations, we found that littermates of the same sex and genetic background exhibited markedly heterogeneous responses to a stroke insult. Our data further supported that variation in the gut microbiota and metabolites contributes to substantial differences in susceptibility to stroke injury, thereby offering mechanistic hypotheses and potential biomarkers for subsequent targeted validation and intervention studies.

Previous studies have reported that taxa including *Clostridiales*, *Clostridia*, *Ruminococcaceae*, and *Oscillospira* are representative butyrate-producing bacteria (29–34). Overall, butyrate-producing bacteria may exert protective effects in neurological and gastrointestinal disorders by maintaining intestinal barrier homeostasis, modulating immune-inflammatory response, and participating in host metabolic regulation (45, 46). In the context of stroke, accumulating evidence indicates that a reduction in butyrate-producing bacteria is associated with more pronounced gut barrier disruption, systemic inflammatory activation, and bad functional outcomes (47, 48). Moreover, emerging studies in post-stroke cognitive impairment demonstrated that restoring butyrate-producing bacteria could improve cognitive function, supporting a potential role for these taxa in post-stroke recovery (49). In our study, LEfSe analysis revealed a significant enrichment of butyrate-producing bacteria in the RES group, suggesting a potential association with better stroke prognosis and further supporting the protective role of butyrate-producing bacteria in post-stroke outcomes.

In this study, KEGG pathway enrichment highlighted glutathione metabolism as a potential contributor to differential stroke vulnerability. Glutathione is central to antioxidant defense, nutrient metabolism, and the regulation of diverse cellular processes in the host (50). In experimental ischemic stroke models, glutathione supplementation has been shown to attenuate cerebral infarction and neuronal cell death (51), and enhancing glutathione biosynthesis (e.g., via γ-glutamylcysteine) can mitigate ischemia-induced neuronal injury (52). Mechanistic studies have implicated glutathione-dependent enzymes, including glutathione peroxidase 4, in limiting lipid peroxidation and ferroptotic cell death after stroke, further supporting the relevance of glutathione metabolism to ischemic vulnerability (53). In line with this concept, our study identified L-glutamic acid, a key substrate for glutathione synthesis, as one of the 11 key differential metabolites in this study. Moreover, our integrated multi-omic analysis showed that the differential genera were significantly correlated with L-glutamic acid. Together, these findings suggest that baseline differences in gut microbial communities may shape a glutamate-glutathione metabolic milieu that strengthens endogenous antioxidant effects, thereby predisposing the host toward an intrinsically more resilient response to ischemic stroke.

Notably, biotin emerged as a microbiota-linked metabolite in our study. Using Spearman’s correlation analysis to interrogate microbiome-metabolome associations, we found that biotin was significantly correlated with multiple differential genera. This relationship is biologically plausible because gut microbes contribute meaningfully to host biotin availability, and microbiota depletion (e.g., antibiotics) can reduce circulating biotin, supporting a microbiota-biotin axis relevant to host metabolism and barrier function (54). Biotin is increasingly recognized as important for intestinal homeostasis because biotin deficiency or impaired intestinal biotin transport increases gut permeability and promotes mucosal inflammation (55). Given the central role of gut barrier dysfunction and systemic inflammation in stroke, microbiota-dependent biotin may represent an additional pathway linking baseline gut ecosystems to ischemic vulnerability. Translational application has also been noted in “biotin-related” neuroprotection in ischemia; for example, the biotin analog 2-iminobiotin has been evaluated clinically for ischemic stroke in the context of large-vessel occlusion treated with thrombectomy (56). Nevertheless, the contribution of biotin to stroke susceptibility and its underlying mechanisms remain to be elucidated. Future studies with targeted metabolomics are required to validate the biotin alteration and clarify its potential functional role.

Several limitations should be acknowledged in this study. First, although distinct microbiota and metabolite compositions were observed between SEN and RES mice, the mechanisms underlying these changes require further investigation. Second, the sampling of SEN and RES mice was not performed at matched time points, and the lack of longitudinal microbiome profiling may confound baseline susceptibility with post-stroke gut remodeling. Third, the contribution of gut microbiota and metabolites to the severity of brain injury and other organ injuries was not investigated. Finally, differences in gut microbiota or metabolites in patients with varying susceptibility to stroke injury were not examined. Future studies should focus on longitudinal analyses and mechanistic investigations to clarify how baseline microbiota influence stroke outcomes and systemic organ injury.

### Conclusions

In summary, variations in the gut microbiota and its metabolites contribute to substantial differences in stroke susceptibility. These findings reveal a previously unrecognized gut-brain axis and highlight the impact of the gut microbiota on ischemic stroke outcomes.

## Supplementary Material

Reviewer comments

## Data Availability

The raw 16S rRNA gene sequencing data have been deposited in the Sequence Read Archive under accession number PRJNA1131626. The raw untargeted metabolomic data and processed peak tables are available in MetaboLights repository under accession MTBLS10583. The metadata for the 16S rRNA gene sequencing and untargeted metabolomic data sets are publicly available on Zenodo at https://zenodo.org/records/20685591.

## SUPPLEMENTAL MATERIAL

Supplemental Material msystems.00149-26-s0001.pdf Supplemental figures and captions for supplemental tables.

Supplemental Tables msystems.00149-26-s0002.xlsx Tables S1–S3.

OPEN PEER REVIEW reviewer-comments.pdf An accounting of the reviewer comments and feedback.

ASM does not own the copyrights to Supplemental Material that may be linked to, or accessed through, an article. The authors have granted ASM a non-exclusive, world-wide license to publish the Supplemental Material files. Please contact the corresponding author directly for reuse.
