# Supplementary material for: Effects of gut microbiota on the susceptibility of ischemic stroke in mice
Source: mSystems. 2026 Jul 10;11(8):e00149-26. doi: 10.1128/msystems.00149-26 (PMC13483375; doi:10.1128/msystems.00149-26)
Supplement: Supplementary file 1 [file msystems.00149-26-s0001.pdf]

Supplementary materials for

Effects of gut microbiota on the susceptibility of ischemic stroke in mice

Guangyan Wu[#], Huidi Wang[#], Qianyi Zhou, Jingxiang Fu, Feng Zhang, Zhuo Duan, Shuo Wang, Jielin Huang, Hongwei Zhou, Zhenchao Ma[*], Yan He[*], Jia Yin[*], Kaiyu Xu[*]

[#] These authors contributed equally to this work.

Corresponding to

*Correspondence: xukaiyu09@smu.edu.cn (K.X.); yinj@smu.edu.cn (J.Y.); yanhe@smu.edu.cn (Y.H.); mazhenchao1986@163.com (Z.M.)

This file includes:

Supplementary Figures S1-S2

Supplementary Tables S1-S3

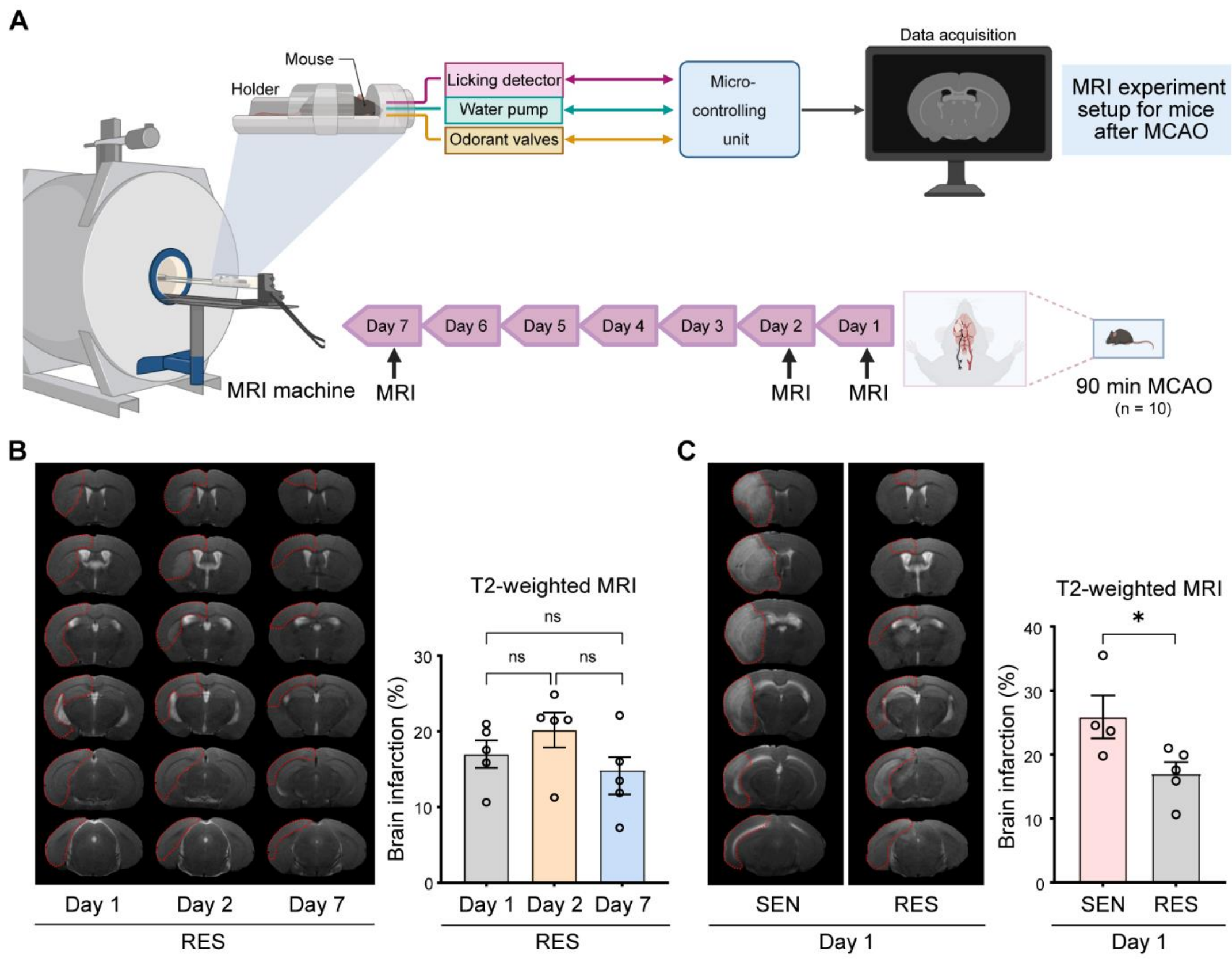

Fig. S1 Longitudinal T2-weighted MRI assessment of brain infarction after 90 min MCAO. (A) Schematic diagram of experimental procedure for MRI scanning in mice subjected to 90 min MCAO. Created with BioRender.com. (B) Time course of infarct volume on T2-weighted MRI at days 1, 2, and 7 after 90 min MCAO in RES mice (n = 5), with representative brain sections and corresponding infarct area. The red dashed lines delineated the infarction region. Data were expressed as mean ± SEM. *P* values were determined by one-way ANOVA, followed by Tukey's test. "ns" indicated "not significant". (C) The representative brain sections on T2-weighted MRI and corresponding infarct area between SEN (n = 4) and RES (n = 5) mice at day 1 after 90 min MCAO. The red dashed lines delineated the infarction region. Data were expressed as mean ± SEM. Statistical analysis was assessed using the two-sided Student's *t* test. *$P < 0.05$. MCAO, middle cerebral artery occlusion; MRI, magnetic resonance imaging; RES, ischemic stroke-resistant; SEN, ischemic stroke-sensitive.

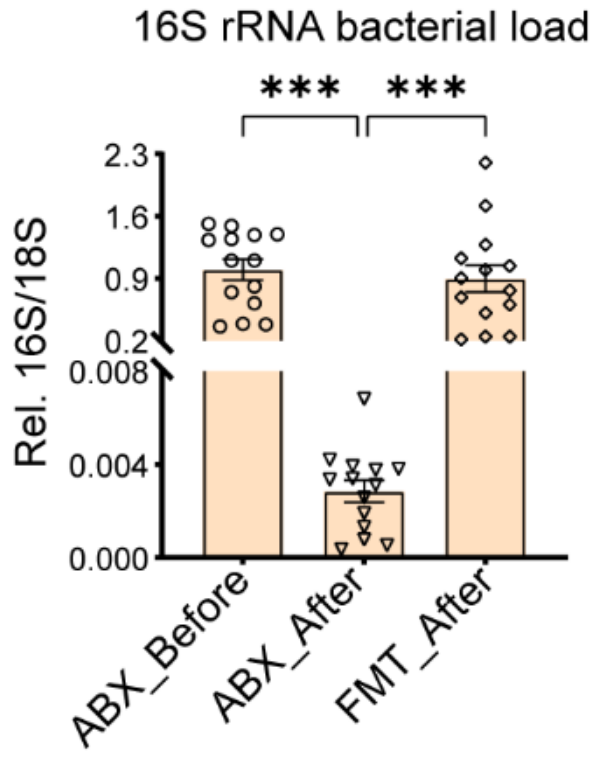

Fig. S2 The relative fecal 16S rRNA bacterial load before ABX, after ABX, and after FMT (n = 14). Data were expressed as mean ± SEM. *P* values were determined by Kruskal-Wallis test, followed by Dunn's multiple comparisons test. ***$P < 0.001$. ABX, broad-spectrum antibiotics; FMT, fecal microbiota transplantation.

Table S1. Sequences of primers. All primer sequences were presented in the 5'-3' orientation.

Table S2. Differential gut microbial taxa identified by LEfSe between the RES and SEN groups. Differential taxa were identified using LEfSe (LDA effect size). *P* values were adjusted for multiple testing using the Benjamini-Hochberg FDR method. "Significance" indicated whether each taxon met the predefined thresholds: LDA > 2.0 and FDR-adjusted *P* value < 0.05 (*) or LDA > 2.0 and FDR-adjusted *P* value < 0.01 (**). FDR, false discovery rate; LEfSe, linear discriminant analysis effect size; LDA, linear discriminant analysis; RES, ischemic stroke-resistant; SEN, ischemic stroke-sensitive.

Table S3. Raw metabolites identified between the RES and SEN groups by untargeted metabolomics. This table presented all metabolites detected in the RES and SEN groups by untargeted metabolomics, including metabolite name, molecular formula, molecular weight, annotation information, detection details, and raw abundance values. Statistical parameters comparing the RES and SEN groups were also provided, including ROC, VIP, FC, $\log_2(FC)$, *P* value, and Benjamini-Hochberg FDR-adjusted *P* value. FC, fold change; FDR, false discovery rate; RES, ischemic stroke-resistant; ROC, receiver operating characteristic; SEN, ischemic stroke-sensitive; VIP, variable importance in the projection.
