# Supplementary material for: Effects of gut microbiota on the susceptibility of ischemic stroke in mice
Source: mSystems. 2026 Jul 10;11(8):e00149-26. doi: 10.1128/msystems.00149-26 (PMC13483375; doi:10.1128/msystems.00149-26)
Supplement: Supplementary file 2 [file reviewer-comments.pdf]

AMERICAN SOCIETY FOR MICROBIOLOGY | mSystems®

Effects of gut microbiota on the susceptibility of ischemic stroke in mice

Guangyan Wu, Huidi Wang, Qianyi Zhou, Jingxiang Fu, Feng Zhang, Zhuo Duan, Shuo Wang, Jielin Huang, Hong-Wei Zhou, Zhenchao Ma, Yan He, Jia Yin, and Kaiyu Xu

Corresponding Author(s): Kaiyu Xu, Southern Medical University

Review Timeline:

Submission Date:	February 15, 2026
Editorial Decision:	April 7, 2026
Revision Received:	April 23, 2026
Accepted:	May 22, 2026

Editor: Promi Das

Reviewer(s): The reviewers have opted to remain anonymous.

Transaction Report:

(Note: With the exception of the correction of typographical or spelling errors that could be a source of ambiguity, letters and reports are not edited. The original formatting of letters and referee reports may not be reflected in this compilation.)

DOI: https://doi.org/10.1128/msystems.00149-26

Re: mSystems00149-26 (**Effects of gut microbiota on the susceptibility of ischemic stroke in mice**)

Dear Prof. Kaiyu Xu:

Thank you for the privilege of reviewing your work. Below you will find my comments, instructions from the mSystems editorial office, and the reviewer comments.

Please return the manuscript within 60 days; if you cannot complete the modification within this time period, please contact me. If you do not wish to modify the manuscript and prefer to submit it to another journal, notify me immediately so that the manuscript may be formally withdrawn from consideration by mSystems.

Revision Guidelines
To submit your modified manuscript, log into the submission site at https://msystems.msubmit.net/cgi-bin/main.plex. Go to Author Tasks and click the appropriate manuscript title to begin. The information you entered when you first submitted the paper will be displayed; update this as necessary. Note the following requirements:

• Upload point-by-point responses to the issues raised by the reviewers in a file named "Response to Reviewers," NOT in your cover letter.
• Upload a compare copy of the manuscript (without figures) as a "Marked-Up Manuscript" file.
• Upload a clean .DOC/.DOCX version of the revised manuscript and remove the previous version.
• Each figure must be uploaded as a separate, editable, high-resolution file (TIFF or EPS preferred), and any multipanel figures must be assembled into one file.
• Any supplemental material intended for posting by ASM should be uploaded with their legends separate from the main manuscript. You can combine all supplemental material into one file (preferred) or split it into a maximum of 10 files with all associated legends included.

For complete guidelines on revision requirements, see our Submission and Review Process webpage. Submission of a paper that does not conform to guidelines may delay acceptance of your manuscript.

Data availability: ASM policy requires that data be available to the public upon online posting of the article, so please verify all links to sequence records, if present, and make sure that each number retrieves the full record of the data. If a new accession number is not linked or a link is broken, provide mSystems production staff with the correct URL for the record. If the accession numbers for new data are not publicly accessible before the expected online posting of the article, publication may be delayed; please contact production staff (mSystems@asmusa.org) immediately with the expected release date.

Publication Fees: For information on publication fees and which article types are subject to charges, visit our website. If your manuscript is accepted for publication and any fees apply, you will be contacted separately about payment during the production process; please follow the instructions in that e-mail. Arrangements for payment must be made before your article is published.

ASM Membership: Corresponding authors may join or renew ASM membership to obtain discounts on publication fees. Need to upgrade your membership level? Please contact Customer Service at Service@asmusa.org.

The ASM Journals program strives for constant improvement in our submission and publication process. Please tell us how we can improve your experience by taking this quick Author Survey.

Thank you for submitting your paper to mSystems.

Sincerely,
Promi Das
Editor
mSystems

Reviewer #1 (Comments for the Author):

I thank the authors for the extensive effort they put into revising their work. While the additional statistical details are helpful, they are insufficient and I have remaining concerns.

1. Incomplete and unclear statistical framework for differential metabolite identification

The manuscript reports differential metabolites based on VIP > 1, fold change thresholds, and FDR-adjusted P-values in Fig 4 legend and line 387; however, the statistical methodology used to generate these P-values is not described. Specifically, it is unclear which statistical test was applied and whether a multiple testing correction procedure was used (which appears to be the case in Table S3). Because PCA and PLS-DA do not provide univariate statistical inference, this represents a critical omission that limits reproducibility and interpretability. A clear and complete description of the statistical testing workflow is required.

In addition, combining supervised PLS-DA-derived VIP scores with inferential P-values for feature selection is sub-optimal and potentially biased, as VIP scores are derived from a model trained to separate the same groups. A more rigorous approach would rely on a well-defined univariate statistical framework for identifying differential metabolites, with multivariate methods used primarily for visualization.

2. Suboptimal application and interpretation of Mantel tests

The use of Mantel tests in the integrative analysis is not optimal. Mantel tests are designed to assess the correlation between two multivariate distance matrices and is intended for global associations (Please see Mangnier et al., A systematic benchmark of integrative strategies for microbiome-metabolome data, Communications Biology 2025). Here, the authors constructed a dissimilarity matrix from only 7 preselected differential genera, and computed Euclidean distance matrices separately for each individual metabolite, followed by multiple Mantel tests. This approach reduces the metabolite data to a series of univariate distance structures and does not fully leverage the multivariate nature of metabolomics data. A more appropriate strategy would be to construct a single distance matrix based on the full set of key metabolites and assess its association with the microbiome distance matrix. Here, the correct interpretation would be that samples that differ in the microbiome also differ in the metabolome, and is currently overinterpreted (Line 421-422).

Given that the Spearman correlation analysis (Fig. 5A) already provides more direct and interpretable relationships between specific genera and metabolites, the Mantel analysis does not appear to add substantial additional insight. I suggest either removing the Mantel analysis or substantially revising its rationale, implementation, and interpretation.

Dear Editor,

We are grateful for the constructive and insightful comments provided on our manuscript entitled "**Effects of gut microbiota on the susceptibility of ischemic stroke in mice**" (Submission ID: mSystems00149-26). Guided by your constructive feedback, we have completed the required revisions to comprehensively address all reviewer concerns. The main revisions in the manuscript and our responses to the editor's and reviewers' comments are as follows:

Reviewer #1 (Comments for the Author):

I thank the authors for the extensive effort they put into revising their work. While the additional statistical details are helpful, they are insufficient and I have remaining concerns.

1. Incomplete and unclear statistical framework for differential metabolite identification

The manuscript reports differential metabolites based on VIP > 1, fold change thresholds, and FDR-adjusted P-values in Fig 4 legend and line 387; however, the statistical methodology used to generate these P-values is not described. Specifically, it is unclear which statistical test was applied and whether a multiple testing correction procedure was used (which appears to be the case in Table S3). Because PCA and PLS-DA do not provide univariate statistical inference, this represents a critical omission that limits reproducibility and interpretability. A clear and complete description of the statistical testing workflow is required.

In addition, combining supervised PLS-DA-derived VIP scores with inferential P-values for feature selection is sub-optimal and potentially biased, as VIP scores are derived from a model trained to separate the same groups. A more rigorous approach would rely on a well-defined univariate statistical framework for identifying differential metabolites, with multivariate methods used primarily for visualization.

Response:

We thank the reviewer for this insightful comment. We agree that our original description of the statistical workflow for differential metabolite identification was incomplete and lacked sufficient detail.

In the revised manuscript, we have clarified the statistical workflow for differential metabolite identification as follows: after data preprocessing and normalization, the relative abundance values of metabolites were $\log_2$-transformed prior to statistical analysis to approximate normal distribution. Each metabolite was individually compared between groups using two-sided Welch's t-test, and the resulting P values were adjusted for multiple comparisons using the Benjamini-Hochberg false discovery

rate (FDR) method. Metabolites with FDR-adjusted $P < 0.05$ and fold change (FC) > 1.5 or FC < 0.667 were considered statistically significant.

PLS-DA-derived variable importance in projection (VIP) scores were used only to prioritize biologically relevant metabolites for downstream interpretation and were not used as criteria for differential metabolite selection. The overall statistical framework applied in this study is consistent with commonly used analytical strategies in metabolomics studies (1-3).

As described above, we confirmed that the data presented in Figure 4D are correct and therefore no modification was required. We have revised the relevant Methods section, the legend for Figure 4D, and the legend of Table S3 to clarify this point and avoid any potential misunderstanding.

The Methods section has been revised as follows: "Differential metabolite analysis was performed as follows. After data preprocessing and normalization, the relative abundance values of metabolites were $\log_2$-transformed prior to statistical analysis to approximate normal distribution. Each metabolite was individually compared between groups using two-sided Welch's *t*-test, and the resulting *P* values were adjusted for multiple comparisons using the Benjamini-Hochberg FDR method. Metabolites with FDR-adjusted $P < 0.05$ and fold change (FC) > 1.5 or FC < 0.667 were considered statistically significant." (Page 8, lines 195-201)

The legend of Figure 4D has been revised as follows: "(D) Differential metabolites between the RES and SEN groups (n =10-13 per group). Volcano plot showing 168 significantly altered metabolites in RES versus SEN group, including 87 up-regulated and 81 down-regulated metabolites. Differential metabolites were defined by FDR-adjusted *P* value < 0.05 and FC > 1.5 or FC < 0.667." (Page 34, lines 863-867)

The legend of Table S3 has been revised as follows: "Table S3. Raw metabolites identified between the RES and SEN groups by untargeted metabolomics. This table presented all metabolites detected in the RES and SEN groups by untargeted metabolomics, including metabolite name, molecular formula, molecular weight, annotation information, detection details, and raw abundance values. Statistical parameters comparing the RES and SEN groups were also provided, including ROC, VIP, FC, $\log_2$(FC), *P* value, and Benjamini-Hochberg FDR-adjusted *P* value. FC, fold change; FDR, false discovery rate; RES, ischemic stroke-resistant; ROC, receiver operating characteristic; SEN, ischemic stroke-sensitive; VIP, variable importance in the projection." (Supplementary materials, Page 6)

These revisions improve the transparency and reproducibility of the statistical framework.

2. Suboptimal application and interpretation of Mantel tests

The use of Mantel tests in the integrative analysis is not optimal. Mantel tests are designed to assess the correlation between two multivariate distance matrices and is

intended for global associations (Please see Mangnier et al., A systematic benchmark of integrative strategies for microbiome-metabolome data, Communications Biology 2025). Here, the authors constructed a dissimilarity matrix from only 7 preselected differential genera, and computed Euclidean distance matrices separately for each individual metabolite, followed by multiple Mantel tests. This approach reduces the metabolite data to a series of univariate distance structures and does not fully leverage the multivariate nature of metabolomics data. A more appropriate strategy would be to construct a single distance matrix based on the full set of key metabolites and assess its association with the microbiome distance matrix. Here, the correct interpretation would be that samples that differ in the microbiome also differ in the metabolome, and is currently overinterpreted (Line 421-422).

Given that the Spearman correlation analysis (Fig. 5A) already provides more direct and interpretable relationships between specific genera and metabolites, the Mantel analysis does not appear to add substantial additional insight. I suggest either removing the Mantel analysis or substantially revising its rationale, implementation, and interpretation.

Response:

We sincerely thank the reviewer for this insightful comment. We agree that the Mantel test is intended for assessing global associations between multivariate distance matrices, and that our original analysis and interpretation were not appropriate.

Following the reviewer's suggestion, we have removed the Mantel analysis from the revised manuscript rather than constructing a global metabolite distance matrix based on the full set of differential metabolites. This revision improves the methodological rigor of the integrative analysis and avoids potential overinterpretation of the association between the gut microbiome and metabolome.

Since the Spearman correlation analysis directly evaluates pairwise associations between differential genera and metabolites and provides clearer biological interpretability at the feature level, we retained this analysis as the main integrative result in Figure 5. The corresponding methods, results, and related discussion have been revised accordingly. (Pages 8-9, lines 212-220; Pages 16-17, lines 420-434; Page 20, lines 523-526)

The revised Figure 5 is provided below for reference.

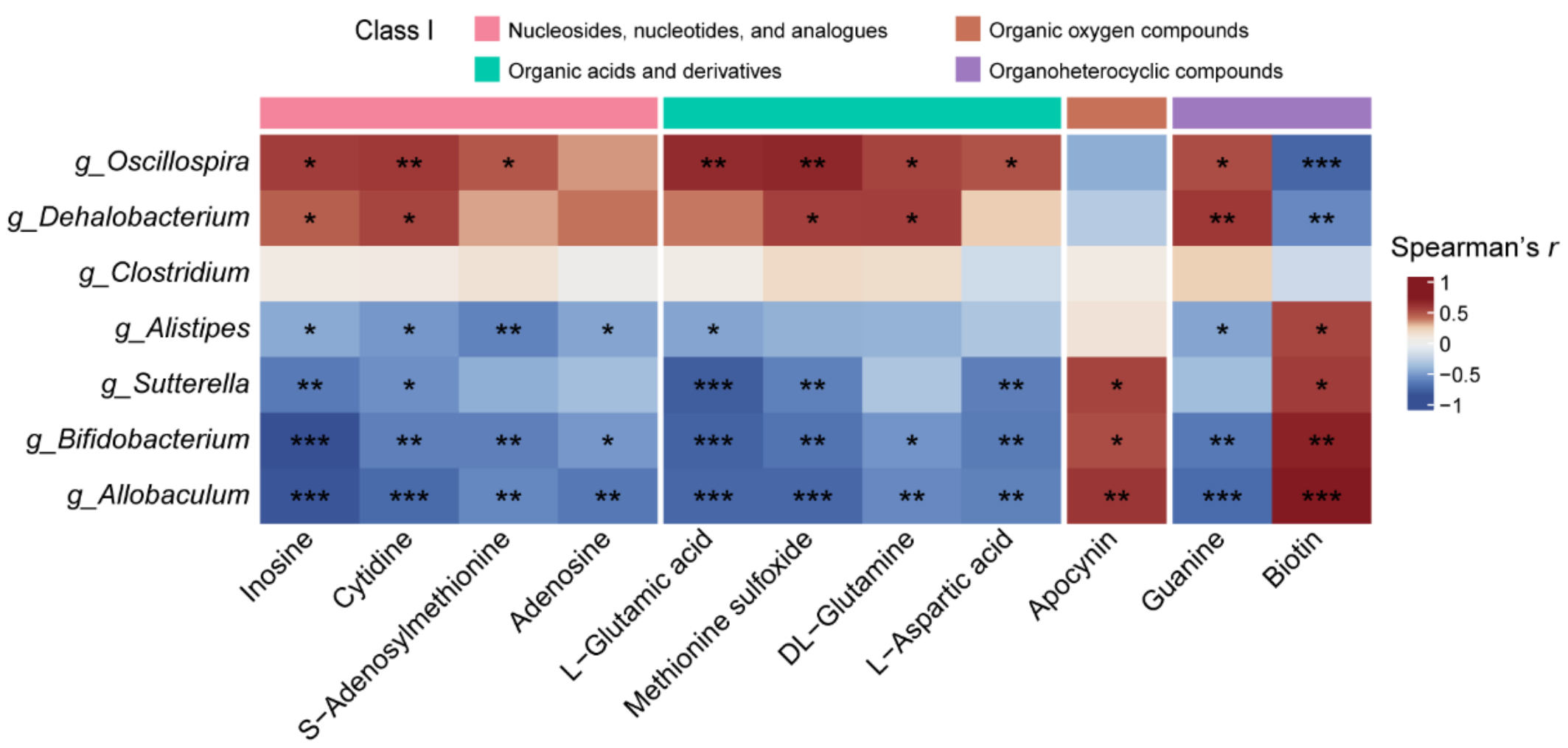

Fig. 5 Correlation analysis between differential gut microbiota and metabolites in cecal contents. Spearman's correlation analysis between the 7 different genera and the 11 key differential metabolites (inosine, cytidine, S-adenosylmethionine, adenosine, L-glutamic acid, methionine sulfoxide, DL-glutamine, L-aspartic acid, apocynin, guanine, and biotin). Red and blue denoted positive and negative correlations, respectively, with color intensity proportional to the correlation coefficient (Spearman's *r*). *P* values were adjusted for multiple testing using the Benjamini-Hochberg FDR method. Significant correlations are marked with asterisks. FDR-adjusted $P < 0.05$ (*), FDR-adjusted $P < 0.01$ (**), FDR-adjusted $P < 0.001$(***). FDR, false discovery rate. (Pages 34-35, lines 879-899)

We sincerely appreciate the constructive suggestions from you and the reviewers. In response to the reviewer's comment, we have carefully revised the manuscript. We look forward to your consideration of the revised manuscript.

With best regards,

Dr. Kaiyu Xu
Microbiome Medicine Center
Department of Laboratory Medicine, Zhujiang Hospital
Southern Medical University, Guangzhou, China
On behalf of all authors

References

1. Cui H, Chen Y, Li K, Zhan R, Zhao M, Xu Y, Lin Z, Fu Y, He Q, Tang PC, Lei I, Zhang J, Li C, Sun Y, Zhang X, Horng T, Lu HS, Chen YE, Daugherty A, Wang D, Zheng L. 2021. Untargeted metabolomics identifies succinate as a biomarker and therapeutic target in aortic aneurysm and dissection. Eur Heart J 42:4373-4385.

2. Lai Y, Liu CW, Yang Y, Hsiao YC, Ru H, Lu K. 2021. High-coverage metabolomics

uncovers microbiota-driven biochemical landscape of interorgan transport and gut-brain communication in mice. Nat Commun 12:6000.

3. Black B, da Silva LBR, Hu G, Qu X, Smith DFQ, Magaña AA, Horianopoulos LC, Caza M, Attarian R, Foster LJ, Casadevall A, Kronstad JW. 2024. Glutathione-mediated redox regulation in Cryptococcus neoformans impacts virulence. Nat Microbiol 9:2084-2098.

Re: mSystems00149-26R1 (**Effects of gut microbiota on the susceptibility of ischemic stroke in mice**)

Dear Prof. Kaiyu Xu:

Your manuscript has been accepted, and I am forwarding it to the ASM production staff for publication. Your paper will first be checked to make sure all elements meet the technical requirements. ASM staff will contact you if anything needs to be revised before copyediting and production can begin. Otherwise, you will be notified when your proofs are ready to be viewed.

Data Availability: ASM policy requires that data be available to the public upon online posting of the article, so please verify all links to sequence records, if present, and make sure that each number retrieves the full record of the data. If a new accession number is not linked or a link is broken, provide production staff with the correct URL for the record. If the accession numbers for new data are not publicly accessible before the expected online posting of the article, publication may be delayed; please contact ASM production staff immediately with the expected release date.

Publication Fees: For information on publication fees and which article types have charges, please visit our website. We have partnered with Copyright Clearance Center (CCC) to collect author charges. If fees apply to your paper, you will receive a message from no-reply@copyright.com with further instructions. For questions related to paying charges through RightsLink, please contact CCC at ASM_Support@copyright.com or toll free at +1-877-622-5543. CCC makes every attempt to respond to all emails within 24 hours.

ASM Membership: Corresponding authors may join or renew ASM membership to obtain discounts on publication fees. Need to upgrade your membership level? Please contact Customer Service at Service@asmusa.org.

Embargo Policy: A press release may be issued as soon as the manuscript is posted on the mSystems Latest Articles webpage. The corresponding author will receive an email with the subject line "ASM Journals Author Services Notification" when the article is available online.

Cover Image Submissions: If you would like to submit a potential Cover Image, please email a file and a short legend to msystems@asmusa.org. Please note that we can only consider images that (i) the authors created or own and (ii) have not been previously published. By submitting, you agree that the image can be used under the same terms as the published article. Image File requirements: TIF/EPS, 7.5 inches wide by 8.25 inches tall (at least 2,250 pixels wide by 2,475 pixels tall), minimum 300 dpi resolution (600 dpi preferred), RGB, and no figure elements, e.g., arrows or panel labels. The legend should be a short description of the image, 1-2 sentences recommended. Please download and use this interactive template in Adobe to ensure that your proposed cover image meets our size requirements (https://journals.asm.org/pb-assets/pdf-text-excel-files/ASM-Interactive-Sizing-Cover-Template-1715689791.pdf).

Thank you for submitting your paper to mSystems.

Sincerely,
Promi Das
Editor
mSystems
